# Association of Household Food Insecurity with the Mental and Physical Health of Low-Income Urban Ecuadorian Women with Children

**DOI:** 10.1155/2016/5256084

**Published:** 2016-09-26

**Authors:** M. Margaret Weigel, Rodrigo X. Armijos, Marcia Racines, William Cevallos, Nancy P. Castro

**Affiliations:** ^1^Department of Environmental Health, School of Public Health, Indiana University Bloomington, Bloomington, IN, USA; ^2^Global Environmental Health Research Laboratory, School of Public Health, Indiana University Bloomington, Bloomington, IN, USA; ^3^Programa Prometeo, Secretaría Nacional de Educación Superior, Ciencia, Tecnología e Innovación, Quito, Ecuador; ^4^Centro de Biomedicina, Facultad de Ciencias Medicas, Universidad Central del Ecuador, Quito, Ecuador; ^5^Departamento de Nutricion Humana, Escuela de Salud Publica, Universidad San Francisco de Quito, Quito, Ecuador

## Abstract

Chronic physical and mental health conditions account for a rising proportion of morbidity, mortality, and disability in the Americas region. Household food insecurity (HFI) has been linked to chronic disease in US and Canadian women but it is uncertain if the same is true for low- and middle-income Latin American countries in epidemiologic transition. We conducted a survey to investigate the association of HFI with the physical and mental health of 794 women with children living in low-income Quito, Ecuador, neighborhoods. Data were collected on HFI and health indicators including self-reported health (SF-1), mental health (MHI-5), blood pressure, and self-reported mental and physical health complaints. Fasting blood glucose and lipids were measured in a subsample. The multivariate analyses revealed that HFI was associated with poorer self-rated health, low MHI-5 scores, and mental health complaints including stress, depression, and ethnospecific illnesses. It was also associated with chest tightness/discomfort/pain, dental disease, and gastrointestinal illness but not other conditions. The findings suggest that improving food security in low-income households may help reduce the burden of mental distress in women with children. The hypothesized link with diabetes and hypertension may become more apparent as Ecuador moves further along in the epidemiologic transition.

## 1. Introduction

Food insecurity, “the limited or uncertain availability of nutritional adequate and safe foods or limited or uncertain ability to acquire acceptable foods in socially acceptable ways,” [1] is reported to be highly prevalent in many low- and middle-income Latin American countries [2–11] including Ecuador [12–15]. Ecuador and many of its neighbors [16] are undergoing nutritional and epidemiologic transitions marked by increasing dietary intakes of energy-dense, ultra-processed fast/convenience foods and beverages, lower physical activity and increased sedentarism, and increasing rates of obesity, diabetes, hypertension, and other chronic diseases [17–20]. The prevalence of depression, anxiety disorders, and other mental health conditions also are reported to be increasing in Ecuador and many other Latin American populations [16, 21–23]. The rising rates of chronic physical and mental conditions, in addition to the substantial burden imposed by vector-borne and other infectious diseases, are placing a severe strain on the already overburdened public health systems and national economies throughout the region [24, 25].

Overnutrition is a well-documented major driver of diabetes, hypertension, and other chronic diseases. The evidence suggests that consumption of energy-dense diets is associated with increased body weight and other adiposity indicators [26]. It has been hypothesized that HFI promotes overnutrition in women vis-à-vis overconsumption of energy-dense processed, snack and convenience foods leading to weight gain and overweight/obesity. However, the evidence from studies supporting this hypothesis is mixed for North American [27–29] and Latin American populations [12, 13, 30–33]. The findings from a number of studies suggest that, compared to their food secure counterparts, US and Canadian women living in food insecure households tend to have poorer physical health as indicated by self-rated health status [34–37], higher prevalence of type 2 diabetes [37–41], and poorer clinical control of diabetes [42–46]. Some studies also have reported a positive association between HFI and hypertension [37, 45, 47], hyperlipidemia [45, 48], heart disease [37], metabolic syndrome [49], high C-reactive protein levels [50], dental disease [51], and other indicators of poorer physical health [28, 34, 35, 37]. Furthermore, it appears that, as HFI severity rises, so does the severity of many adverse health outcomes [52] and that the relationship between HFI and poor health is bidirectional [52, 53].

The experience of being food insecure is stressful. It has been hypothesized that the reported relationship of HFI with type 2 diabetes and other chronic diseases may be caused by the overproduction of stress hormones as well as other factors including excessive consumption of energy-dense foods, overweight/obesity, constrained eating, reduced basal metabolism, and decreased physical activity. These biobehavioral mechanisms have been reviewed in detail by prior authors [28, 45, 52, 53].

The only published study to examine the association of HFI with chronic diseases in a Latin American population was reported by Pérez-Escamilla and associates [54]. Their multivariate analysis of nationally representative survey data from Mexico indicated that the prevalence of type 2 diabetes and hypertension was significantly increased among food insecure compared to food secure women. However, due to the dearth of published studies, it is uncertain whether the same pattern exists for HFI and chronic diseases in low-income women from other Latin American countries such as Ecuador. It cannot be automatically assumed that it does since although many Latin American populations share some common cultural characteristics (e.g., language and religion), they are often quite different from each other with respect to their food traditions and diet, lifestyles, the environmental conditions in which they live, genetic admixtures, and other population attributes. In addition, they are at various stages in the nutritional and epidemiologic transitions. Such differences have the potential to influence the hypothesized relationship of HFI with chronic disease and other adverse health outcomes.

The association of HFI with mental health indicators has not yet been investigated in Ecuador. However, prior studies conducted in the US [55–59], Canada [40, 52], and several African countries [60–65] have consistently linked HFI to indicators of poorer mental health in women including increased stress, depression, anxiety, and psychological distress. Similar to diet- and stress-sensitive chronic diseases (e.g., hypertension and diabetes), the relationship between HFI and mental health in women appears to be dose-response and mostly likely of a bidirectional nature [52, 53, 66, 67]. It has been reported that psychological distress and other mental health problems may mediate the relationship between HFI and physical health outcomes such as poorer sleep quality [68, 69]. In addition, HFI can not only directly influence women's mental and physical health and well-being but also, in the case of maternal caretakers, may contribute to poorer parenting and, ultimately, poorer child physical and mental health, growth and developmental delays, and other adverse outcomes [56, 70, 71].

The major aim of the present study was to examine the association of HFI with general and specific physical and mental health indicators in women with minor children living in low-income neighborhoods in a large urban center in Ecuador. We hypothesized that HFI, especially the more severe level (i.e., very low food security), would be associated with poorer self-rated overall health, physical and mental health complaints and conditions, and clinical and laboratory indicators of chronic disease especially diabetes, hypertension, and hyperlipidemia. We based our hypotheses on reviews of the body of evidence from US and Canadian studies, the single Latin American (Mexican) study published by Pérez-Escamilla and associates [54], and conceptual frameworks proposed by prior authors explain the relationship of HFI with physical and mental health outcomes [27–29, 45, 52, 53].

## 2. Methods

This study was part of larger investigation of the food, nutrition, and health issues of adult women and their minor children living in low-income neighborhoods in Quito, the capital city of Ecuador. We previously reported on the association of HFI with diet and other nutritional status indicators in the same sample of adult women [12]. Details on participant selection, informed consent, and general methodology have been previously reported elsewhere [12]. Briefly, the 794 women participants and their children were recruited using nonprobabilistic convenience sampling. This was done through public schools and community health centers located in five low-income Quito neighborhoods during May–August in 2010–2012 and 2014. As we also previously reported [12], study site and participant selection was informed by our community outreach work with local partners from public elementary schools and public health clinics located in low-income Quito neighborhoods. One women from each household participated in the face-to-face interviews and other assessments. Prospective participants were eligible for study inclusion if they were the mother or another adult female head of household (e.g., grandmother, aunt, and stepmother) living in the same home with at least one schoolchild (ages 6–12 years), a permanent resident of their present community, and did not have any conditions that would make it difficult for them to understand or reply to interview questions. The study protocol was approved by institutional review boards at the University of Texas at El Paso and Central University of Ecuador, Biomedical Research Center. The women participants gave their written informed consent prior to beginning the study. They were given oral and written interpretations of their clinical, laboratory, anthropometric, and other screening results and, where indicated, were provided with written referrals for follow-up care through the free Ecuadorian public health or social security health systems.

The food security status of participant households was assessed using a language-adapted version of the US Household Food Security Survey Module (HFSSM), hereafter referred to as the Quito HFSSM. The experience-based instrument consisted of 18 multiple choice questions covering a wide range of household experiences and behaviors reported for the prior 12 months. Affirmative answers to the 18 questions in the scale were used to construct raw scores for the households which were then classified as food secure or food insecure (i.e., low food security or very low food security). Households with low food security were characterized by decreased dietary quality, variety, or desirability but little or no indication of reduced food intake. Those with very low food security reported decreased food intakes as well as multiple indicators of disrupted eating [12]. As we previously reported, we examined the internal validity and other psychometric properties of the Quito HFSSM [12]. Our analyses confirmed that the instrument's performance was consistent with the theoretical framework of household food insecurity as a managed process and it exhibited good validity and reliability [12].

Data on participant, household, and neighborhood characteristics were collected during face to-face interviews. These included age, education, marital status, pregnancy status, occupation, current employment, monthly household* per capita* income, place of birth, location of current neighborhood residence, residence length in current neighborhood, and family size.

The SF-1 general health question from the Short Form 36 (SF-36) was used to assess self-reported general health status during the past 12 months. This item has been used extensively by surveys to assess the general health and well-being of persons from general populations or those with specific health problems [72]. Possible responses on the 5-point Likert scale ranged from “excellent” to “poor.” Participant answers were dichotomized as “fair-to-poor” or “other” in the analyses to allow for comparison with prior studies. Question 2 on the SF-36 assessed the self-rated health of participants compared to the previous 12 months. The possible responses on the 5-point scale ranged from “much better” to “much worse” [73]. Participant responses on this question were also were dichotomized, in this case as “worse-to-much worse” versus “other.”

With the Mental Health Inventory (MHI-5), the mental health subscale of the SF-36 was used to measure general mental health status. Possible scores on the scale ranged from 0 to 100 with lower scores indicating greater psychological distress or worse mental health. The conservative cut-off point of 52 was used for the MHI-5 in this study which is often used to identify individuals at high risk for severe depression or other mental health problems [74]. The validity and reliability of both the MHI-5 and the larger SF-36 instrument have been previously validated in Ecuadorian immigrants [75] and other Latin American and Spanish-speaking groups [76]. Cronbach's alpha for the MHI-5 [0.72] was in the acceptable coefficients range of 0.65–0.8 [77].

We used our previously developed structured health questionnaire containing closed- and open-ended questions to elicit information from participants on specific physical and mental health complaints for the past 12-month period such as diabetes, hypertension, heart disease respiratory diseases (e.g., asthma and chronic bronchitis), allergies, neurological problems (e.g., migraine/chronic headaches and epilepsy), dental disease, eye/vision problems, and gastrointestinal illnesses (e.g., diarrhea, nausea, vomiting, or stomach pain lasting 3 or more continuous days in a single week) [78, 79]. If participants reported answers in the affirmative, they were then asked to further describe the particular health complaint.

The same questionnaire also was used to query participants as to whether or not they had experienced depression, anxiety, stress, or any ethnospecific illness symptoms (e.g., nervios, corajes, and sustos/espantos) during the previous 12 months and, if so, to further describe the problem in detail [78, 79]. Ethnospecific illnesses are culturally defined conditions that form part of traditional belief systems. These explanatory mechanisms for illness, many of which represent cultural concepts of psychological distress, are common in Ecuador and throughout Latin America [78–80]. Two of these, “nervios” and “sustos/espantos,” are formally recognized in the DSM-5 [80]. “Nervios” is a chronic stress-related condition characterized by anxiety and desperation manifested as agitation, irritation, crying, and insomnia. “Sustos/espantos” is believed to be caused by frightening event resulting in sleep difficulties, malaise, appetite disturbances, and gastrointestinal complaints. “Corajes” (i.e., frequent angry outbursts caused by frustration and anger) and “latidos” (i.e., rapid heart palpitations caused by stress, anxiety, or fright) are examples of other ethnospecific conditions common throughout the region [78, 80].

Blood pressure was measured from the right arm of seated participants using a calibrated automatic sphygmomanometer with an adjustable cuff (Omron Health Care, IL). Measurements were performed after participants had rested quietly for 15 minutes. Systolic and diastolic pressure were measured to the nearest 2 mmHg and the average of the two was recorded. The classification of participant blood pressure and hypertension risk was made following the Joint National Committee on Prevention, Detection, Evaluation, and Treatment of High Blood Pressure, 7th Report recommendations [81].

Fasting blood glucose and lipid profiles were obtained from a consecutive sample of 269 or 96% of the women participants from the 2014 data collection period. We were not able to obtain blood samples on 10 participants at one study site because of a technical error. A fingertip capillary blood sample was used to measure fasting blood glucose and lipids (i.e., total cholesterol, LDL, HDL, and triglycerides). The analyses were performed with the Cholestech LDX Analyzer according to manufacturer specifications [Alere, Waltham, MA]. The instrument was calibrated prior to each screening session. Criteria established by the American Diabetes Association [82] and National Cholesterol Education Program [83] were used, respectively, to classify the fasting blood glucose and lipid measurements used for the analyses. These were high blood glucose (>126 mg/dL), high total cholesterol (>240 mg/dL), high LDL (>160 mg/dL), low HDL (<50 mg/dL), and high triglycerides (>200 mg/dL).

The anthropometric characteristics of the women participants were described in a previous publication [12]. We describe the measurement methods again in the present work since BMI was included as a covariate in the statistical analyses and abdominal obesity was used as one of the indicators of possible metabolic syndrome. Briefly, participant weights were measured using a calibrated scale (Detecto, Webb City, MO) and their heights measured using a stadiometer (Seca NA, Chino, CA). The weight and height measurements were used to calculate body mass index (BMI) defined as weight (kg)/height (m)^2^ [84]. Waist circumference was obtained using a semiflexible anthropometry measuring tape (Seca NA, Chino, CA) following previously reported measurement methods [12]. Women with a waist circumference of ≥88 cms were classified as having abdominal obesity. Those with three or more of the following indicators or those taking antihypertensive or glucose or cholesterol-lowering medications classified with metabolic syndrome using established criteria [85]. These included systolic blood pressure (≥130 mmHg) or diastolic blood pressure (≥85 mmHg), fasting blood glucose (≥100 mg/dL), fasting blood triglycerides (≥150 mg/dL) and cHDL (<50 mg/dL), and waist circumference ≥88 cm.

### 2.1. Data Analysis

The data were analyzed with IBM-SPSS statistical analysis software (version 23, IBM Corp.). The unadjusted analyses used Poisson regression with robust variance estimation to examine the association of household food security status (food secure, low food security, and very low food security) with health indicators. They included the SF1 (fair-poor health versus other), SF2 (worse-to-much worse health), MHI-5 score (≤52 versus >52), and a number of individual physical and mental health-related complaints (presence versus absence) which had a prevalence of ≥2% in the sample. This same analytic method also was used to investigate the association of HFI with multiple health complaints (≥3 versus <3). The subsequent multivariate analyses used the same analytical method with adjustments made for factors previously identified as associated with HFI in our prior study including household* per capita* monthly income, participant education, and participant residence length in their current Quito neighborhood [12]. Additional adjustments were made for participant age, current neighborhood site, data collection year, and BMI.

The association of household food security status with mean between-group differences in measured blood pressure, fasting blood glucose, and lipids was analyzed using general linear models (GLM). The subsequent GLM multivariate models adjusted for the same covariates mentioned above. In this study, *p* values <0.05 were considered as statistically significant.

## 3. Results

The characteristics of the 794 women participants were described in detail in a prior publication [12]. Briefly, 81% reported that their households had been food insecure in the past 12 months; 41% reported low food security and 40% very low food security. The participants were relatively young (average age: 34 ± 11 years). Sixteen (2%) reported being pregnant at the time of their interview. Most participants were in a legal (29.6%) or in a common law marital union (39.3%) and lived in households averaging approximately 4.6 ± 1.6 persons. Slightly more than half were full-time housewives (53%) and most had only a middle school education or less (8 ± 3.8 years). The* per capita *monthly income of households averaged US$ 110 ± 104. Forty-four percent reported that they had been born in Quito. Sixty-one of participants reported being long-term residents of their current Quito neighborhood (i.e., ≥50% of lifetime). Their current residences were located within the Cotocollao (32%), El Dorado (27%), Chillogallo (20%), El Camal (13%), and Los Chillos (8%) neighborhood sectors.

Table 1 shows the findings from the regression models indicating that participants from the most severely affected households, that is, those with very low food security, were more likely than those from food secure homes to self-rate their current health as being only “fair-to-poor.” They also were more likely to rate it as being “worse-to-much worse” than in the 12 months prior to the interview.

Table 2 displays the results of the unadjusted and adjusted regression analysis results indicating that women from households with very low food security were more likely to have a low MHI-5 score suggestive of poorer mental health status and being at risk for severe depression or other adverse mental health outcomes compared to those who were food secure.

Table 3 displays the regression analysis results showing the association of household food security status with participant-reported mental and physical health complaints. As it indicates, both the unadjusted and adjusted analyses indicate that participants from households with very low food security were more inclined than food secure women to report feelings of depression, stress, or an ethnospecific illness, especially “corajes” or “nervios,” during the previous 12-month period. They also were more likely to report having experienced chest pain/discomfort/tightness, gastrointestinal symptoms lasting three or more continuous days in a single week, and dental disease. In contrast, food insecure participants were not more likely to report having diabetes, hypertension, or other health conditions.

As we reported previously [12], based on their BMIs, 1% of the nonpregnant women in the sample were classified with underweight, 38% with normal weight, and 61% with either overweight (38%) or obesity (23%). Fifty-three percent also had waist circumferences greater than 88 centimeters indicating abdominal obesity. However, the proportion of participants with either generalized overweight/obesity or abdominal obesity did not differ by their household food security status [12].

Table 4 displays the findings from the unadjusted and adjusted GLM analyses investigating the association of household food security status with measured clinical and laboratory health indicators. The unadjusted analysis results showed that women from households with very low food security had average systolic blood pressure (SBP) and diastolic blood pressure (DBP) that were increased by four mmHg over those of women from food secure and low food security households. However, the contribution of household food security status to SBP and DBP was no longer apparent in the subsequent analyses which adjusted for covariates. Nearly 17% of the participants had blood pressure suggestive of possible hypertensive disease (SBP > 140 mmHg or DBP > 90 mmHg) but no statistically significant differences were identified in the proportion with high blood pressures from food secure versus food insecure households in either the unadjusted or adjusted regression analyses (data not shown).

Table 4 also displays the findings from the fasting blood glucose and lipid measurements made on the participant subsample. As shown, the mean unadjusted and adjusted fasting blood glucose and lipid concentrations did not differ by the household food security status of participants. Eight percent had fasting blood glucose concentrations exceeding 126 mg/dL but the proportion with high glucose values did not differ by household food security status in either the unadjusted or adjusted regression analyses (data not shown). The proportion identified with abnormal blood lipid values was as follows: high (3.7%) and borderline high (22.3%) total blood cholesterol, high (3.7%) and borderline high LDL (13.4%), low HDL (59.5%), and high triglycerides (17.8%). However, no measureable differences were found in the unadjusted nor adjusted regression analyses regarding the proportion of participants with abnormal blood lipid values from the three household food security groups (data not shown). Finally, close to one-quarter (21.6%) of the participants were positive for three or more measured indicators suggestive of possible metabolic syndrome but proportion of these was not different between those from food insecure and food secure households (data not shown).

## 4. Discussion

This study is the first to report on the association of HFI with the mental and physical health of an Ecuadorian group. It also is one of a small handful of published studies to examine the association of HFI with the mental or physical health outcomes of adults living in Latin American countries. As we reported previously [12], the high prevalence of food insecurity reported by the Ecuadorian households was consistent with estimates published for low-income urban households in other Latin American countries using similar experience-based instruments. The major findings from this study were that very low food security was associated with fair-to-poor self-rated health, multiple health complaints, poorer mental health, poorer dental health, gastrointestinal illness, and chest tightness/discomfort/pain. However, it was not associated with self-reported nor clinical indicators of hypertension, diabetes, heart disease, or other increasingly more prevalent chronic diseases in Ecuador [17–19].

The study data were consistent with our hypothesis that food insecure women, especially those from more severely affected households, would be more likely than those that were food secure to report poorer mental health. We are unable to compare our study findings on the association of HFI with self-rated health with women from other groups in Ecuador or Latin America because of a lack of published studies. However, our results are consistent with those published on an African LIMC [86] and populations in the US [34, 35] and Canada [36, 37]. This is important since the evidence suggests that women who self-rate their health as only fair-to-poor are more likely to have depression, functional impairment, and poorer well-being than those with higher self-ratings [87]. The findings on the poorer self-rated health of more severely affected food insecure participants were supported by other study data including lower MHI-5 scores, self-reported depression, stress, and ethnospecific indicators of psychological distress, in this case, “corajes” and “nervios.” We note that although “corajes” and “nervios” have different behavioral manifestations, both are believed to result from exposure to external stressors [79, 80] in this case, food insecurity. Another potential evidence of HFI-linked stress comes from the finding that women from more severely affected food insecure homes were also more likely to complain of chest tightness/discomfort/pain. Such somatic symptoms are reported as common in women suffering from stress and anxiety [88]. Indeed, this could be a likely explanation since our post hoc analysis results did not find an association between this complaint and self-reported (i.e., hypertension, heart disease, asthma, and bronchitis) or clinical cardiorespiratory (i.e., high blood pressure and hyperlipidemia) in this relatively young sample of women.

Our findings generally concur with other studies conducted in other LIMCS [60–65] and in the US [55–59] and Canada [40, 52] linking HFI to poorer mental health in women as manifested by symptoms of stress, depression, anxiety, and psychological distress. The experience of living in a food insecure household is stressful and particularly so for mothers with dependent children. Several potential pathways exist by which HFI may promote or exacerbate mental health problems such as was the case for the women in our study. Those living in impoverished households are often forced to make difficult decisions about whether to spend their scarce resources on food or on other necessities (e.g., housing, utilities, child care, educational expenses, clothing, and health care). This type of stressful decision-making can promote frustration, anxiety, feelings of helplessness, and symptoms of depression. In addition, the cross-cultural evidence strongly suggests that women often sacrifice food quality and diversity in order to stretch scarce household food resources [1, 27, 53]. This coping strategy may be effective in reducing physiological hunger in the immediate term but, over time, can result in inadequate dietary intakes of iron, vitamin A, B-complex vitamins, and other essential nutrients needed to maintain good mental and physical health. Furthermore, if food insecurity continues to worsen, mothers often skip meals or reduce their own food portions in order to buffer their children from hunger [1, 27, 53]. Such buffering behavior may be beneficial for children but may increase the risk for hypoglycemia and its physical manifestations such as nervousness, irritability, and/or angry outbursts [89, 90] similar to what the women in our study reported.

The evidence from our study strongly suggests, but does not prove, that very low food security promotes psychological distress and other adverse mental health outcomes in low-income Ecuadorian women with children. However, it is also possible that preexisting depression, anxiety disorders, or other forms of psychological distress, in either the women participants, their children, or other household members, may have increased overall household vulnerability to food insecurity. Such conditions could impede women's ability to obtain or maintain stable employment, shop around for good quality food at lower prices, or prepare meals because of the cost and time required for self-care or care-taking of affected family members or health-seeking [43, 44, 46, 52]. Whatever the mechanism, poor maternal mental health manifested as depression, anxiety, or other forms of psychological distress is a well-documented risk factor for suboptimal parenting practices. This is important since the children of mothers affected by HFI appear to be at risk for poorer health, growth and developmental delays, and behavioral and academic problems [59, 91–93].

Different from our* a priori* hypothesis, we were unable to confirm an association between HFI and either diabetes or hypertension. Our findings differ from the only other Latin American country study (Mexico) which found diabetes and hypertension to be more prevalent among food insecure compared to food secure women [53]. They also differ from prior studies conducted in low-income US and Canadian women linking HFI to a higher diabetes prevalence [37–41] and, to a lesser extent, hypertension and certain other chronic diseases and their risk factors [37, 45, 47–50].

The reason for the differences between studies regarding HFI and chronic disease is not immediately evident but may be due to population differences related to nutritional status, age, ways of coping with stress, or other unreported factors. For example, different from that reported for Latin American countries further along in their nutritional and epidemiologic transitions, that is, Mexico and Brazil [30–32], HFI was not associated with dietary or anthropometric indicators of overnutrition in this [12] or other Ecuadorian adult female groups [13–15]. This is important point since one of the primary pathways through which HFI is hypothesized to promote the development of diabetes, hypertension, and similar chronic diseases is through the excessive consumption of energy-dense foods, constrained eating, and obesity [28, 29, 40, 52]. Another reason may be because the adverse effects of excess body adiposity had not yet become apparent in this group of mostly premenopausal mothers in their mid-thirties. However, it is possible that if Ecuador continues along on the same nutritional and epidemiologic transition trajectory, then in the future, a similar pattern of association between HFI, diabetes, and other chronic disease may become manifest.

Dental disease in adults is associated with poorer diet, inflammation, and higher risk for chronic diseases including diabetes, hypertension, cardiovascular disease, peripheral arterial disease, and stroke [94]. The prevalence of dental disease in Ecuador is reported to be among the highest in the Latin America region [95]. In our study, HFI was associated with poorer self-reported caries, gum disease, and other poorer dental health indicators in Ecuadorian women, a finding consistent with findings reported for US [51, 96] and Brazilian adults and children [97, 98]. The evidence from this low-income sample [12] and other Ecuadorian groups [13, 14] indicates that women from food insecure households do not consume more sweets nor other caries-promoting foods compared to their more food secure counterparts [12, 13] although their consumption of fruits, vegetables, and other groups high in micronutrients and other anti-inflammatory food constituents was significantly reduced. It is uncertain whether diet, other behaviors (e.g., tooth brushing), cost and other dental health care access barriers, and/or other factors are responsible for the observed association. The potential role of HFI in dental disease among adults is understudied and needs to be further explored due to its important nutrition and health implications.

In this study, women living in more severely affected food insecure households were more likely to report experiencing one or more episodes of diarrhea, nausea, vomiting, or stomach pain lasting three or more continuous days in a single week. This finding concurs with those previously reported for Mexican immigrant households [78]. Such symptoms are most likely due to gastrointestinal infection caused by eating microbe-contaminated foods and/or poorer gut immunity associated with undernutrition but could also reflect somatization of mental distress.

The potential limitations of this work need to be taken into account when interpreting its findings. For example, the cross-sectional design permits inference but cannot be used to establish temporal or causal effects regarding HFI and health outcomes. Although the study findings suggest that HFI promotes poorer mental health in women, it is also possible that preexisting mental health conditions could have influenced the household vulnerability to food insecurity. In addition, the relationship between HFI and mental health is most likely bidirectional whereby poorer mental health distress is both a cause and a consequence of HFI [52]. Future studies should employ longitudinal designs to identify the directionality of associations.

Since the study used a nonprobabilistic sampling method, the women participants and their households may not necessarily be representative of those from other low-income urban neighborhoods in Quito. Thus, care should be taken in extrapolating the results to other urban groups. Another potential limitation relates to the timing of data collection. This was dictated by the research team's availability at the study site. We also point out that the measurement of household food security status was limited to the 12-month period prior to participant interviews so it might not necessarily reflect the food situation of a household over a longer period of time. In addition, food may not always be equitably distributed within households, so it is possible that the food security status of a household may not necessarily reflect that of women residing within a home.

Another potential limitation regards the use of self-reported health complaints which rely on participant memory. However, we restricted the timing of these to the past 12 months and we also collected additional objective clinical (blood pressure), anthropometric, and laboratory health indicators (fasting blood glucose and lipid profile) following standard protocols. In the case of the latter, these were from a subsample; they may not be representative of all study participants. In addition, the potential for type 2 error exists since the power could have been too low to detect a significant difference in the association of HFI with blood glucose and lipids among the subsample of 269 participants if one existed. The possibility also exists for confounding by being unaccounted for variables that accentuate or suppress the association between HFI and the health variables measured in this study.

Despite its potential limitations, the present study adds to the limited literature reporting on the relationship of HFI with the mental and physical health of low-income women living in Latin American countries in various stages of nutritional and epidemiologic transition. The findings suggest that improving food security status of the households of low-income Ecuadorian women has the potential to also improve their mental and physical health. Household food insecurity is a potentially modifiable factor that can be reduced through improving food social safety nets, women's educational and employment opportunities, financial and nutrition literacy, and other multipronged strategies to increase access to low-cost, nutritious food and enhance their food and other household resource management skills. As we previously reported [12], the Ecuadorian government has passed laws assuring citizen rights to food security, food sovereignty, adequate nutrition, good health, and a number of other social protections. Ecuador's* Buen Vivir *(Collective Good Living) National Development Plan has implemented specific priorities, goals, and action plans and increased national expenditures on social safety net program for the purpose of reducing poverty rates, access to food, and improving population health and well-being [12]. However, it is not possible to accurately assess whether policies and programs intended to improve population food security are having the intended impact without performing systematic HFI surveillance. Experience-based food security scales, such as the one used in this study, may help to improve food security governance [99]. Thus, we recommend that the Ecuadorian government undertake systematic surveillance similar to that currently used by the US, Canada, and several other countries in the Americas region. Ideally, a household and/or individuals food security scales could be added to national health and nutrition surveys such as the 2012* Encuesta Nacional de Salud y Nutricion* (Ecuadorian National Health and Nutrition Survey) [17], other existing population surveys, and/or the national census conducted by the* Instituto Nacional de Estadísticas y Censos* (National Institute of Statistics and Census-INEC).

## Figures and Tables

**Table 1:** Association of household food insecurity with self-rated health status (*n* = 794).

Self-rated health status	Food secure (*n* = 152) number (%)	Low food security (*n* = 325) number (%)	Very low food security (*n* = 317) number (%)	Low food security		Very low food security	
				Unadjusted PR (95% CI)^a,b^	Adjusted PR (95% CI)^a,b^	Unadjusted PR (95% CI)^a,b^	Adjusted PR (95% CI)^a,b^
Current health: fair-to-poor	39 (25.7)	112 (34.5)	210 (66.2)	1.34 (0.99, 1.83)	1.22 (0.90, 1.65)	2.58 (1.95, 3.42)^*∗*^	2.09 (1.56, 2.80)^*∗*^

Health compared to 12 mos. ago: worse-to-much worse	27 (17.8)	53 (16.3)	100 (31.5)	0.92 (0.60, 1.40)	1.00 (0.67, 1.53)	1.78 (1.22, 2.59)^*∗∗*^	1.92 (1.27, 2.86)^*∗∗∗*^

^a^PR: prevalence ratio; 95% CI: 95% confidence interval.

^b^Analyses adjusted for participant age, education, *per capita* monthly income, long-term neighborhood residence (≥50% of lifetime), BMI, residence neighborhood, and data collection year.

^*∗*^ *p* = 0.0001; ^*∗∗*^ *p* = 0.003; ^*∗∗∗*^ *p* = 0.002.

**Table 2:** Association of household food security status with low mental health inventory-5 (MHI-5) score.

	Food secure (*n* = 152) number (%)	Low food security (*n* = 325) number (%)	Very low food security (*n* = 317) number (%)	Low food security		Very low food security	
				Unadjusted PR (95% CI)^a,b^	Adjusted PR (95% CI)^a,b^	Unadjusted PR (95% CI)^a,b^	Adjusted PR (95% CI)^a,b^
Low MHI-5 score (≤3)	29 (19.2)	88 (27.6)	136 (43.0)	1.44 (1.00, 2.08)	1.40 (0.96, 2.03)	2.24 (1.58, 3.18)^*∗*^	2.16 (1.50, 3.11)^*∗*^

^a^PR: prevalence ratio; 95% CI: 95% confidence interval.

^b^Analyses adjusted for participant age, education, *per capita* monthly income, long-term neighborhood residence (≥50% of lifetime), BMI, residence neighborhood, and data collection year.

^*∗*^ *p* = 0.0001.

**Table 3:** Association of household food insecurity with self-reported mental and physical health complaints (*n* = 794)^a^.

Self-reported health complaints	Food secure (*n* = 152) number (%)	Low food security (*n* = 325) number (%)	Very low food security (*n* = 317) number (%)	Low food security		Very low food security	
				Unadjusted PR (95% CI)^b^	Adjusted PR (95% CI)^b,c^	Unadjusted PR (95% CI)^b^	Adjusted PR (95% CI)^b,c^
Depression	19 (12.5)	64 (19.7)	88 (27.8)	1.72 (0.99, 2.98)	1.78 (1.10, 3.13)	2.69 (1.57, 4.62)^*∗*^	2.64 (1.48, 4.72)^*∗*^
Stress	8 (5.3)	20 (6.2)	36 (11.4)	1.17 (0.53, 2.59)	1.23 (0.56, 2.72)	2.16 (1.04, 4.53)^∧^	2.14 (1.01, 4.65)^∧^
≥1 ethnospecific illnesses	35 (23.0)	87 (26.8)	134 (42.3)	1.16 (0.83, 1.64)	1.11 (0.78, 1.56)	1.84 (1.33, 2.52)^*∗*^	1.68 (1.20, 2.33)^*∗∗∗*^
(i) Corajes	15 (9.9)	42 (12.9)	63 (19.9)	1.03 (0.97, 1.10)	1.03 (0.97, 1.10)	1.11 (1.04, 1.18)^#^	1.12 (1.04, 1.21)^*∗∗∗*^
(ii) Nervios	9 (5.9)	22 (6.8)	39 (12.3)	1.01 (0.96, 1.06)	0.99 (0.95, 1.05)	1.07 (1.01, 1.12)^#^	1.05 (1.00, 1.11)^∧^
(iii) Sustos/espantos	4 (2.6)	19 (5.8)	17 (5.4)	2.22 (0.77, 6.42)	2.27 (0.75, 6.85)	2.04 (0.70, 5.95)	2.00 (0.63, 6.40)

Chest pain/discomfort/tightness	8 (5.3)	19 (5.8)	39 (12.3)	1.11 (0.50, 2.48)	1.29 (0.59, 2.83)	2.34 (1.12, 4.88)^*∗∗*^	3.04 (1.42, 6.50)^*∗∗*^
Hypertension	10 (6.6)	26 (8.0)	23 (7.3)	1.22 (0.60, 2.46)	1.22 (0.60, 2.49)	1.10 (0.54, 2.26)	1.01 (0.48, 2.11)
Cardiovascular disease	29 (19.1)	68 (20.9)	103 (32.5)	1.10 (0.74, 1.62)	1.04 (0.70, 1.54)	1.70 (1.18, 2.45)^*∗∗*^	1.38 (0.93, 2.05)
Diabetes	8 (5.3)	17 (5.2)	12 (3.8)	0.99 (0.44, 2.25)	0.38 (0.06, 2.64)	0.72 (0.30, 1.72)	0.66 (0.09, 4.65)
Chronic respiratory conditions	31 (20.4)	62 (19.1)	89 (28.1)	0.94 (0.64, 1.38)	0.92 (0.62, 1.35)	1.38 (0.96, 1.97)	1.37 (0.94, 2.00)
Gastrointestinal illnesses	41 (27.0)	105 (32.3)	146 (46.1)	1.20 (0.88, 1.63)	1.22 (0.90, 1.66)	1.71 (1.28, 2.28)^*∗*^	1.77 (1.31, 2.38)^*∗*^
Allergies	32 (21.1)	60 (18.5)	81 (25.6)	0.88 (0.60, 1.29)	1.36 (0.82, 2.23)	1.21 (0.85, 1.74)	1.50 (0.83, 2.68)
Migraine, chronic headaches	7 (4.6)	18 (5.5)	19 (6.0)	1.20 (0.51, 2.82)	1.00 (0.38, 2.64)	1.30 (0.56, 3.03)	1.74 (0.71, 4.27)
Vision	377 (47.5)	62 (41.3)	150 (46.4)	1.14 (0.85, 1.51)	1.18 (0.94, 1.48)	1.27 (0.92, 1.75)	1.29 (0.99, 1.66)
Dental problems	82 (54.3)	204 (62.8)	234 (73.8)	1.16 (0.98, 1.38)	1.14 (0.96, 1.35)	1.37 (1.17, 1.61)^*∗*^	1.30 (1.10, 1.54)^#^

≥3 complaints	67 (44.1)	164 (50.5)	206 (65.0)	1.39 (0.72, 2.68)	1.11 (0.89, 1.40)	2.90 (1.42, 5.95)^*∗*^	1.48 (1.19, 1.85)^*∗*^

^a^Self-reported complaints with a prevalence of 2% or more in sample.

^b^PR: prevalence ratio; 95% CI: 95% confidence interval.

^c^Analyses adjusted for participant age, education, *per capita* monthly income, long-term neighborhood residence (≥50% of lifetime), BMI, residence neighborhood, and data collection year.

^*∗*^ *p* = 0.0001; ^*∗∗*^ *p* = 0.004; ^*∗∗∗*^ *p* = 0.003; ^∧^ *p* = 0.042; ^#^ *p* = 0.002.

**Table 4:** Association of household food security status with fasting blood glucose and lipid levels: participant subsample (*n* = 269).

	Food secure (*n* = 58)		Low food security (*n* = 121)		Very low food security (*n* = 90)		ANOVA *p* value	ANCOVA *p* value^a^
	Mean ± SD	Adjusted mean^a^	Mean ± SD	Adjusted mean^a^	Mean ± SD	Adjusted mean^a^		
*Blood pressure*								
Systolic blood pressure (mmHg)	117 ± 15	118	117 ± 15	118	121 ± 17	119	0.001	0.77
Diastolic blood pressure (mmHg)	72 ± 12	74	72 ± 13	73	76 ± 15	74	0.0001	0.32

*Fasting blood glucose* (mg/dL)	87 ± 9	86	89 ± 21	89	85 ± 9	85	0.09	0.13

*Fasting blood lipid profile *								
Total cholesterol (mg/dL)	183 ± 30	181	179 ± 40	179	177 ± 30	178	0.57	0.86
Low-density lipoprotein (mg/dL)	105 ± 30	104	99 ± 33	100	96 ± 30	100	0.26	0.41
High-density lipoprotein (mg/dL)	49 ± 15	50	46 ± 13	46	51 ± 16	50	0.07	0.07
Triglycerides (mg/dL)	128 ± 54	120	158 ± 102	159	136 ± 65	140	0.05	0.06

^a^Analyses adjusted for participant age, education, *per capita* monthly income, long-term neighborhood residence (≥50% of lifetime), BMI, residence neighborhood, and data collection year.
